# Primary Vesical Actinomycosis in a 23-Year-Old Man

**DOI:** 10.1155/2014/691360

**Published:** 2014-06-17

**Authors:** Tevfik Ziypak, Senol Adanur, Fatih Ozkaya, Muhammet Calık, Ozkan Polat, Yilmaz Aksoy, Isa Ozbey

**Affiliations:** ^1^Department of Urology, Faculty of Medicine, Ataturk University, 25240 Erzurum, Turkey; ^2^Department of Pathology, Faculty of Medicine, Ataturk University, 25240 Erzurum, Turkey

## Abstract

*Introduction*. Actinomycosis can affect any organ of the body, although cutaneous fistulas are common in actinomycotic infections, and other organs such as the bladder are only rarely involved. *Case Presentation*. Herein we report and discuss a young male patient with primary vesical actinomycosis. A 23-year-old man was hospitalized complaining of intermittent gross hematuria over a 6-month duration. The patient underwent a cystoscopic examination under general anesthesia; an edematous, hyperemic, wide-based mass, which protruded from the dome of the bladder, was seen and incompletely resected. The histopathological examination of the material showed *Actinomyces* organisms surrounded by inflammation and a photomicrograph showed the microorganism. After confirmation of bladder actinomycosis, the patient received penicillin. A CT scan of the abdomen and pelvis showed no evidence of the mass at the postoperative 6th month. Cystoscopic examination showed complete healing of the transurethral resection area at the dome of the bladder. *Conclusion*. In conclusion, we believe that the gold standard treatment for vesical actinomycosis should include the combination of a transurethral resection of the mass and long-term penicillin treatment.

## 1. Introduction

Human actinomycosis is generally caused by* Actinomyces israelii* and was first described in 1878 by Israel; however, at least six species of* Actinomyces* are pathogenic to humans.* Actinomyces israelii* is gram-positive, filamentous anaerobic, or microaerophilic bacterium normally found in the mouth and colon, but it is an opportunistic pathogen causing disease only in previously damaged tissue [1].* Actinomyces* species are pleomorphic and branching filamentous bacteria that are currently classified in the genus* Actinomyces* of the order Actinomycetales, although they were once thought to be fungi because of their branching filaments [2]. Actinomycosis usually occurs in immunocompetent persons but may also occur in persons with immunodeficiency. Actinomycosis can affect any organ of the body, although cutaneous fistulas are common in actinomycotic infections. Cervicofacial, thoracic, abdominopelvic, and cerebral actinomycosis comprise the majority of human actinomycosis cases. Abdominal actinomycosis may spread into the pelvis; however, pelvic actinomycosis is usually seen in women and is secondary to the insertion of intrauterine devices (IUD). Vesical actinomycosis usually occurs via direct extension from primary infection of the pelvic organs and primary vesical actinomycosis is a very rare disease.

*Actinomyces* species are susceptible to several antimicrobials including penicillin G, chloramphenicol, tetracyclines, erythromycin, clindamycin, imipenem, streptomycin, and cephalosporins. Penicillin G is the main drug used regardless of the severity and location of actinomycosis. Fluoroquinolones, aztreonam, fosfomycin, and other aminoglycosides generally have poor activity against* Actinomyces* species [3]. For penicillin-allergic patients, tetracyclines, macrolides, and lincomycin are appropriate options. Parenteral penicillin G, 10–20 million U/d divided every 6 hours, should be administered for 4–6 weeks, followed by oral penicillin V, 2–4 g/d divided every 6 hours for 6–12 months [4]. Ordinarily, actinomycosis in the cervicofacial region can be efficiently treated with a 2-month course of an oral antimicrobial agent without the need for surgical resection. However, more severe and complicated cases should be managed with combined surgical resection and high dosage and prolonged antibiotic treatment.

Herein, we report and discuss the case of a young male patient with primary vesical actinomycosis in terms of history, symptomatology, laboratory and radiological findings, treatment modalities, and follow-up.

## 2. Case Report

**Figure 1:**
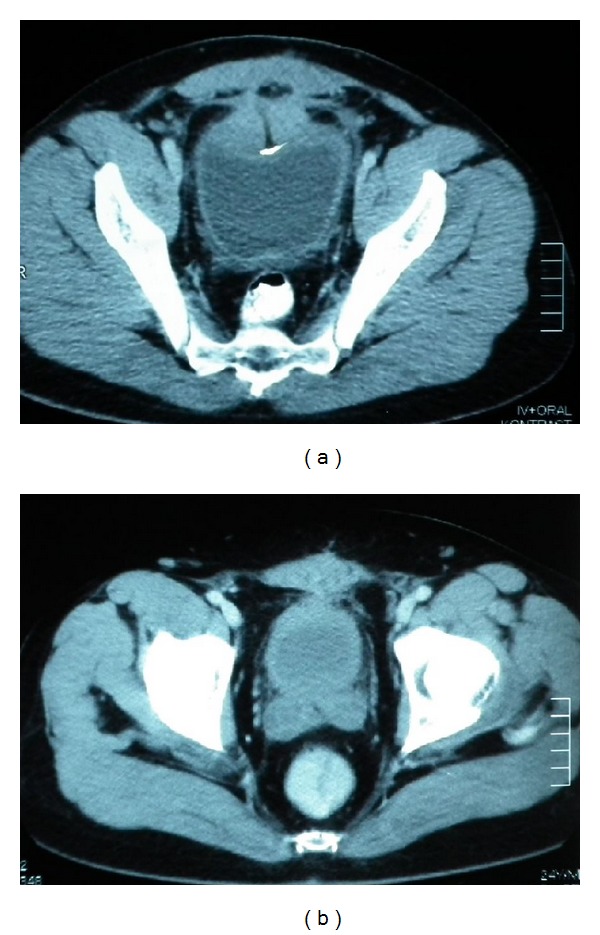
(a) A computed tomography (CT) scan of the abdomen and pelvis showing a mass lesion, involving the dome of the urinary bladder, invading the rectus fascia and muscles. (b) CT scan of the abdomen and pelvis showing no evidence of the mass at the postoperative 6th month.

A 23-year-old man was hospitalized complaining of intermittent gross hematuria over a 6-month duration. He had previously undergone a left nephrectomy due to hydronephrotic atrophy. Additionally, he had undergone an unsuccessful cystoscopy and open bladder surgical attempt due to scar formation in the perivesical area in another hospital. The physical examination was unremarkable and urine analysis showed abundant erythrocytes without leukocytes. The urine culture was sterile and urine cytology showed no evidence of malignant cells. Intravenous urography showed a filling defect on the dome of the bladder. A computed tomography (CT) scan of the abdomen and pelvis showed a mass lesion of about 5 × 4 × 3 cm in size, involving the urinary bladder dome and invading the rectus fascia and muscles (Figure 1(a)).

**Figure 2:**
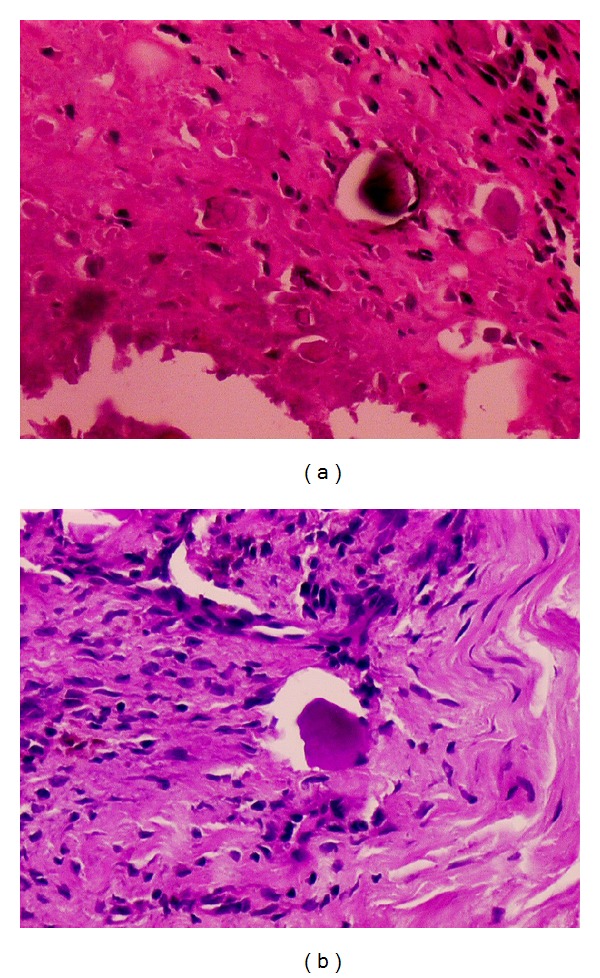
(a) Microscopically, there are a few neutrophils with some* Actinomyces* microorganisms and eosinophilic hyaline material (H&E, 200x). (b) Histochemically, the microorganisms are positive with PAS staining (PAS, 200x).

The patient underwent a cystoscopic examination under general anesthesia; an edematous, hyperemic, wide-based mass, which protruded from the dome of the bladder, was seen and incompletely resected. The histopathological examination of the material showed* Actinomyces* microorganisms surrounded by inflammation (H&E, 200x, Figure 2(a); periodic acid Schiff, 200x, Figure 2(b)).

After confirmation of bladder actinomycosis, the patient received treatment as follows: penicillin at 20 million U/day intravenously for 2 weeks and then 6 million U/day intramuscularly for 4 weeks, followed by ampicillin at 1,500 mg/day orally for a total period of 4 months. A CT scan of the abdomen and pelvis showed no evidence of the mass 6 months postoperatively (Figure 1(b)). Cystoscopic examination showed complete healing of the transurethral resection area in the bladder dome.

## 3. Discussion

The incidence of genitourinary actinomycosis is very low. However, actinomycosis of the kidney, bladder, and scrotum has been previously reported [5–7]. Pelvic actinomycosis is a diagnostic enigma due to the atypical nature of clinical symptoms. The diagnosis of pelvic actinomycosis was rarely made before surgical exploration in all of the previously reported cases [8]. A definitive diagnosis is usually made postoperatively following histopathological evaluation [9]. In tissue sections with hematoxylin-eosin staining, sulfur granules are seen as round, oval, or horseshoe-shaped basophilic masses with a radiating fringe of eosinophilic clubs. Special stains such as Gomori methenamine-silver stain, p-aminosalicylic acid, MacCallen-Goodpasture, or Brown-Brenn stain may be needed [2]. We have applied periodic acid Schiff staining in addition to hematoxylin-eosin. There are only a few reported cases of diagnosis being made before surgical exploration by needle biopsy [10]. Some indications may be useful in the early recognition of this disease, including the prolonged use of an IUD. Patients with an IUD should alert clinicians to consider pelvic actinomycosis in their differential diagnosis and perform a biopsy before surgical exploration.

Vesical actinomycosis usually occurs via direct extension from primary infection of the pelvic organs. Imaging of abdominal and pelvic actinomycosis does not typically help with diagnosis since the disease usually presents as an abscess or a mass lesion and is commonly mistaken for a tumor. Diagnosis of vesical actinomycosis is often delayed due to the possibility of an urothelial malignancy carrying greater indices; initial diagnosis is often difficult and it is usually misdiagnosed as an urothelial or urachal tumor. In our case, the CT indicated a bladder tumor. In vesical actinomycosis, cystoscopy and endoscopic biopsy are recommended. We believe that an endoscopic biopsy is superior to a needle biopsy since it can be carried out under direct visualization. However, a biopsy does not allow the confirmation of radiologic diagnosis in other organs such as the kidney. Actinomycosis can cause hydronephrosis. Many hydronephrosis cases due to actinomycosis infections were reported in the literature [11, 12]. In our case, the patient had undergone a left nephrectomy caused by hydronephrotic atrophy at another hospital. Since we could not obtain the histopathological results of the resected kidney, we concluded that the nephrectomy was likely caused by renal actinomycosis.

Primary bladder actinomycosis can be misdiagnosed as a bladder tumor and may be treated by partial or radical cystectomy. Lim et al. performed partial cystectomy in a patient with urachal actinomycosis since they could not rule out malignancy despite the use of preoperative PET and CT [11]; they reported that their PET/CT findings showed similarities between malignancy and actinomycosis [13]. Therefore, we conclude that all bladder masses should be initially resected transurethrally and only after histopathological confirmation should radical surgical attempts be considered. However, there are a few reports of partial or radical cystectomy in patients with bladder actinomycosis [14, 15].

The contribution of urine analyses is limited in the diagnosis of vesical actinomycosis. Pyuria and microscopic hematuria are found in the urine analysis and urine culture is usually sterile; there are only a few reported cases diagnosed by urine analyses [16, 17]. Jang et al. diagnosed vesical actinomycosis by urine cytology in a female patient [18]; however, they had to verify their diagnosis by endoscopic biopsy and histopathological evaluation. Consequently, we have observed that histopathological diagnosis was made for most of the reported vesical actinomycosis cases in the literature.

Surgery is indicated for the resection of necrotic tissue, excision of sinus tracts, and drainage of empyemas or abscesses. It is believed that surgical resection reduces the total treatment period to 3–6 months [4]. Although surgery can be effective, it is not curative without antimicrobial treatment. Actinomycosis usually shows good prognosis with a low mortality if timely diagnosis is made and appropriate treatment is given. However, long-term follow-up after treatment is essential since relapse is common. Although disseminated disease is uncommon, it could be fatal, even if correctly diagnosed and treated [19, 20]. We believe that the gold standard treatment for vesical actinomycosis should include the combination of transurethral resection of the mass and long-term penicillin treatment.

## 4. Conclusion

Preoperative diagnosis of primary bladder actinomycosis is difficult due to the nature of its clinical presentation, and it can be misdiagnosed as a bladder tumor or treated as bladder cancer. In many instances, it is misdiagnosed as a vesical or urachal tumor and is usually diagnosed postoperatively through pathological confirmation. The true diagnosis of actinomycosis may be missed because of the rarity of this condition. Therefore, actinomycosis cases should be reported in order to increase the awareness of this disease.
